# Developmental Emergence of Phenotypes in the Auditory Brainstem Nuclei of *Fmr1* Knockout Mice

**DOI:** 10.1523/ENEURO.0264-17.2017

**Published:** 2017-12-27

**Authors:** Sarah E. Rotschafer, Karina S. Cramer

**Affiliations:** Department of Neurobiology and Behavior, University of California, Irvine, CA 92697

**Keywords:** auditory, brainstem, fragile x, lateral superior olive, medial nucleus of the trapezoid body, ventral cochlear nucleus:

## Abstract

Fragile X syndrome (FXS), the most common monogenic cause of autism, is often associated with hypersensitivity to sound. Several studies have shown abnormalities in the auditory brainstem in FXS; however, the emergence of these auditory phenotypes during development has not been described. Here, we investigated the development of phenotypes in FXS model [*Fmr1* knockout (KO)] mice in the ventral cochlear nucleus (VCN), medial nucleus of the trapezoid body (MNTB), and lateral superior olive (LSO). We studied features of the brainstem known to be altered in FXS or *Fmr1* KO mice, including cell size and expression of markers for excitatory (VGLUT) and inhibitory (VGAT) synapses. We found that cell size was reduced in the nuclei with different time courses. VCN cell size is normal until after hearing onset, while MNTB and LSO show decreases earlier. VGAT expression was elevated relative to VGLUT in the *Fmr1* KO mouse MNTB by P6, before hearing onset. Because glial cells influence development and are altered in FXS, we investigated their emergence in the developing *Fmr1* KO brainstem. The number of microglia developed normally in all three nuclei in *Fmr1* KO mice, but we found elevated numbers of astrocytes in *Fmr1* KO in VCN and LSO at P14. The results indicate that some phenotypes are evident before spontaneous or auditory activity, while others emerge later, and suggest that Fmr1 acts at multiple sites and time points in auditory system development.

## Significance Statement

Individuals with fragile X syndrome (FXS) are hypersensitive to sound and show enhanced cortical responses to sound stimuli. Recent work suggests that functional and physiologic abnormalities in the auditory brainstem may contribute to the dysfunction found in the auditory system. We investigated the emergence of the FXS auditory brainstem phenotype in the development of auditory brainstem nuclei in FXS model mice (*Fmr1* KO). We found that some of the reductions in cell size and imbalances in inhibitory/excitatory input present in adult *Fmr1* KO mice emerged early in postnatal development. This study suggests that even before hearing onset, loss of the *Fmr1* gene drives disruptions in the auditory system.

## Introduction

Fragile X syndrome (FXS) results from transcriptional silencing of the *FMR1* gene and reduced expression of fragile X mental retardation protein (FMRP; Bailey et al., 1998; O’Donnell and Warren, 2002). Individuals with FXS often display communication disorders (Fidler et al., 2007; Finestack et al., 2009), repetitive behaviors (Feinstein and Reiss, 1998; Belser and Sudhalter, 2001; Baranek et al., 2005), and unusual social interactions (Hagerman et al., 1986); accordingly, FXS is the leading single-gene cause of inherited autism. FXS is also associated with hypersensitivity to sensory stimuli (Miller et al., 1999), especially auditory stimuli (Frankland et al., 2004; Roberts et al., 2005; Gothelf et al., 2008; Hessl et al., 2009; Yuhas et al., 2011). Dysfunction in FXS is evidenced by childhood temporal lobe seizures (Musumeci et al., 1991, 1999; Incorpora et al., 2002), exaggerated auditory cortical responses to sound (St Clair et al., 1987; Rojas et al., 2001; Castrén et al., 2003; Knoth and Lippé, 2012; Van der Molen et al., 2012a, b; Schneider et al., 2013; Knoth et al., 2014), and failure to habituate to sounds (Van der Molen et al., 2012a, b).

In addition to these cortical phenotypes, recent studies suggest that some aspects of auditory dysfunction in FXS arise in the auditory brainstem, particularly in nuclei that comprise the sound localization circuits. Auditory input from the periphery first contacts the cochlear nucleus, and axons from the ventral cochlear nucleus (VCN) send excitatory input to the ipsilateral lateral superior olive (LSO) and the contralateral medial nucleus of the trapezoid body (MNTB). In addition to the large calyx of Held excitatory input from VCN, MNTB also receives glycinergic and GABAergic inhibitory input from the ventral nucleus of the trapezoid body (VNTB; Albrecht et al., 2014). MNTB provides glycinergic inhibitory input to ipsilateral LSO, and the balance of excitation and inhibition in LSO is a primary cue used in the computation of interaural level differences, which are used to estimate sound source locations (Moore and Caspary, 1983; Kuwabara and Zook, 1992).

Many symptoms of FXS have been successfully modeled in *Fmr1* KO mice. *Fmr1* KO mice display auditory cortical hyperexcitability (Gibson et al., 2008; Rotschafer and Razak, 2013) and impaired synchronicity (Gibson et al., 2008; Hays et al., 2011; Lovelace et al., 2016). Behaviorally, *Fmr1* KO mice exhibit audiogenic seizures (Musumeci et al., 2000, 2007; Chen and Toth, 2001), fail to habituate to sound (Lovelace et al., 2016), and fail to attenuate acoustic startle (Chen and Toth, 2001; Nielsen et al., 2002; Frankland et al., 2004). FMRP is prominently expressed in the auditory brainstem nuclei (Beebe et al., 2014; Wang et al., 2014; Zorio et al., 2017), and *Fmr1* KO mice have anatomic and physiologic anomalies within the auditory brainstem (Kulesza and Mangunay, 2008; Kulesza et al., 2011; Lukose et al., 2011). Adult *Fmr1* KO mice have heightened auditory brainstem response (ABR) thresholds, indicating a modest peripheral hearing loss (Rotschafer et al., 2015). In *Fmr1* KO mice, there is an imbalance of excitatory and inhibitory synaptic proteins in the brainstem, with a relative increase in inhibitory inputs to the MNTB and an increase in inputs to the LSO (Rotschafer et al., 2015; Garcia-Pino et al., 2017). Additionally, like their human counterparts (Kulesza and Mangunay, 2008), adult *Fmr1* KO animals have smaller cells within their auditory brainstem nuclei (Rotschafer et al., 2015; Ruby et al., 2015).

The observed *Fmr1* KO phenotypes in the auditory brainstem might result indirectly from reduced peripheral input. Alternatively, they could arise from loss of FMRP in the brainstem nuclei themselves. Here, we examined the developmental emergence of auditory brainstem phenotypes in *Fmr1* KO mice. We found differences in cell size at early postnatal ages. In addition, we found that the imbalances in inhibitory and excitatory synaptic markers in MNTB were evident before hearing onset, and before synaptic increases in LSO were present.

FMRP is expressed in microglia and astrocytes (Gholizadeh et al., 2015), which drive aspects of neuronal dysfunction in FXS. We investigated the emergence of glia in the auditory brainstem. We found that microglia emerge normally in the auditory brainstem in *Fmr1* KO mice. However, we found significantly more astrocytes in VCN and LSO in *Fmr1* KO mice. Additionally, the number of astrocytes in LSO correlated with synaptic markers in *Fmr1* KO mice. Together, these studies demonstrate that several of the auditory brainstem phenotypes in *Fmr1* KO mice arise during early postnatal development, and suggest that FMRP acts at multiple points along the auditory pathway.

## MATERIALS AND METHODS

### Animals

In this study, we used seven postnatal day 1 (P1), eight P6, and fifteen P14 FVB strain wild-type mice, and five P1, fourteen P6, and sixteen P14 *Fmr1* KO mice. To test for possible sex differences, we compared data from a subset of the P14 animals including seven female and eight male wild-type mice and nine female and seven male *Fmr1* KO mice. All procedures were approved by the University of California–Irvine Institutional Animal Care and Use Committee.

### Immunofluorescence

Mice were perfused transcardially with 4% paraformaldehyde (PFA) in PBS, and brains were dissected. Brainstems were fixed in PFA solution for 2 h, then equilibrated in a 30% sucrose solution in PBS overnight. Brains were cryosectioned at 16 μm in the coronal plane at the level of the auditory brainstem nuclei. Sections were mounted on chrome-alum glass slides in a 1-in-4 (P1) or 1-in-6 (P6 and P14) series. Mounted sections were then surrounded with a Pap Pen hydrophobic barrier and rinsed in PBS for 10 min. For antigen retrieval, sections were incubated with 0.1% SDS in PBS solution for 5 min. Slides were rinsed three times in PBS, then incubated with either normal goat blocking solution (4% normal goat serum, 0.1% Triton X-100 in PBS), or bovine serum albumin blocking solution (4% bovine serum albumin, 0.4% Triton X-100 in PBS) for 1 h in a humid chamber at room temperature. Bovine serum albumin blocking solution was used for synaptic marker immunolabeling, and normal goat blocking solution was used for immunolabeling of glial cells. Primary antibodies (see below) were applied, and slides were incubated overnight in a humid chamber. Slides were washed in PBS and incubated at room temperature for 1 h with appropriate Alexa Fluor (Invitrogen) secondary antibodies diluted 1:500. Slides were rinsed three times in PBS and coverslipped with Glycergel mounting medium (Dako C0563).

### Primary antibodies

To detect excitatory synaptic input, we used a guinea pig polyclonal antibody that recognized vesicular glutamate transporter-2 protein (VGLUT2) diluted to 1:2500 (Millipore AB2251). This protein reliably labels calyces of Held and shows stable expression levels during postnatal development in rats (Billups, 2005). Inhibitory synaptic input was examined using a rabbit polyclonal antibody that recognized vesicular GABA transporter protein (VGAT), which is expressed in both GABAergic and glycinergic synaptic terminals (Dumoulin et al., 1999), at a dilution of 1:200 (Phosphosolutions 2100-VGAT). In addition, we performed immunofluorescence for synaptophysin 1, a vesicular membrane protein present in both excitatory and inhibitory presynaptic terminals, to evaluate total synaptic input. We used a guinea pig polyclonal antibody at a 1:500 dilution (Synaptic Systems 101 004). To detect microglia, we used a rabbit polyclonal antibody (Wako 019-19741) generated against a synthetic peptide corresponding to the C terminus of ionized calcium-binding adaptor molecule-1 (Iba1) at a dilution of 1:500. To detect astrocytes, we used a mouse monoclonal antibody (Abcam ab56777) that recognizes aldehyde marker dehydrogenase 1 family L1 (ALDH1L1) at a dilution of 1:200.

### Fluorescent Nissl analysis

To visualize cell bodies and brainstem nuclei, we performed fluorescent Nissl staining using the BrainStain Imaging Kit (Life Technologies B34650). Slides were rinsed in 0.2% Triton X-100 solution in PBS then incubated for 20 min at room temperature with NeuroTrace 530/615 red fluorescent Nissl stain diluted 1:300 in PBS. Slides were then rinsed three times with PBS and coverslipped in Glycergel mounting medium.

To evaluate brainstem nucleus metrics, VCN, MNTB, and LSO of fluorescent Nissl stained sections were imaged at 20× using a Zeiss Axioskop-2 microscope. To study cell size, 40× *z*-stacks of VCN, medial MNTB, lateral MNTB, and LSO were generated. Ten cells were randomly selected from each image for measurement; only cells in which the nucleus was visible and with a cross-sectional area >45 µm^2^ were included in the analysis. For brainstem nucleus size, nuclei from both the right and left hemispheres were imaged, with a minimum of two sections imaged per brain. Nuclei were outlined and measured using AxioVision software. The number of neurons in each section within the outlined nucleus was counted using ImageJ cell counter using above inclusion criteria, and a mean number of cells per section was obtained.

### Synaptic protein analysis

High magnification images (63×) of regions within VCN, medial MNTB, lateral MNTB, and LSO of VGLUT- and VGAT-immunolabeled sections were acquired. Regions of interest (ROIs) within each image were selected. ROI size for each brainstem nucleus was consistent across images: VCN = 10138.8 μm^2^, MNTB = 11799.3 μm^2^, medial MNTB = 6899.5 μm^2^, lateral MNTB = 11,799.3 μm^2^, and LSO = 13,239.5 μm^2^. The red (VGLUT) and green (VGAT) color channels for each ROI were separated, and the threshold of each channel was adjusted to highlight the immunopositive areas within each ROI. ImageJ was used to sum the area of all of the immunolabeled objects within an ROI. Fractional coverage was defined as the summed area of immunolabeling divided by the area of the ROI. To compare the relative amounts of VGLUT and VGAT, we defined a synaptic protein index (I_SP_) to describe each ROI, where I_SP_ = (VGLUT – VGAT)/(VGLUT + VGAT). I_SP_ values range between –1 and 1, with negative values indicating relatively more VGAT, and higher positive values indicating relatively more VGLUT.

### Analysis of microglia and astrocytes

Based on fluorescent Nissl staining, VCN, MNTB, and LSO were outlined using AxioVision software. The outlines of these nuclei were then superimposed on images of Iba1- or ALDH1L1-immunolabeled sections. The ImageJ cell counter function was used to quantify Iba1- or ALDH1L1-positive cells. As both microglia and astrocytes are highly ramified, glia were counted only if the somata were present within a section.

### Statistical methods

We used Sigma Stat and Sigma Plot software for our statistical analyses and graphing. We tested for differences between genotypes (wild-type or *Fmr1* KO) and differences between ages (P1, P6, or P14) using two-way ANOVAs to analyze cell size, nucleus size, number of cells, synaptic protein expression, and number of glia. We also examined medial versus lateral differences in MNTB cell size. We performed separate two-way ANOVAs to test the effects of genotype (wild-type or *Fmr1* KO) and location (medial or lateral MNTB). Statistics supporting data shown in all figures are summarized in Table 1. Pairwise comparisons were made using the Holm-Šídák test. Additional comparisons were made using Pearson correlations along with appropriate Bonferroni corrections to determine whether effects were similar in the three auditory brainstem nuclei examined and whether synaptic proteins were correlated with glial cell numbers.

**Table 1.** Statistical analysis

Figure	Test	Sample size (*n*)	Test statistics	*p*	Power α = 0.050
1*B*	Two-way ANOVA	WT: P1 = 5, P6 = 8, P14 = 15; KO: P1 = 5, P6 = 10, P14 = 16	Age *F* = 74.469; genotype *F* = 2.714; age × genotype *F* = 1.251	Age *p* < 0.001; genotype *p* = 0.105; age × genotype *p* = 0.294	Age α = 1.000; genotype α = 0.236; age × genotype α = 0.085
2*B*	Two-way ANOVA	WT: P1 = 7, P6 = 8, P14 = 10; KO: P1 = 5, P6 = 9, P14 = 10	Age *F* = 234.948; genotype *F* = 56.277; age × genotype *F* = 3.471	Age *p* < 0.001; genotype *p* < 0.001; age × genotype *p* = 0.040	Age α = 1.000; genotype α = 1.000; age × genotype α = 0.460
3*D*	Two-way ANOVA	WT: P1 = 5, P6 = 8, P14 = 15; KO: P1 = 5, P6 = 10, P14 = 16	Location *F* = 10.762; genotype *F* = 240.432; location × genotype *F* = 11.572	Location *p* < 0.001; genotype *p* < 0.001; location × genotype *p* = 0.040	Location α = 0.859; genotype α = 1.000; location × genotype α = 0.887
3*E*	Two-way ANOVA	WT: P1 = 5, P6 = 8, P14 = 15; KO: P1 = 5, P6 = 10, P14 = 16	Location *F* = 14.142; genotype *F* = 39.594; location × genotype *F* = 5.922	Location *p* < 0.001; genotype *p* < 0.001; location × genotype *p* = 0.020	Location α = 0.958; genotype α = 1.000; location × genotype α = 0.577
3*F*	Two-way ANOVA	WT: P1 = 5, P6 = 8, P14 = 15; KO: P1 = 5, P6 = 10, P14 = 16	Location *F* = 144.012; genotype *F* = 36.820; location × genotype *F* = 1.056	Location *p* < 0.001; genotype *p* < 0.001; location × genotype *p* = 0.308	Location α = 1.000; genotype α = 1.000; location × genotype α = 0.053
4*B*	Two-way ANOVA	WT: P1 = 7, P6 = 8, P14 = 10; KO: P1 = 5, P6 = 9, P14 = 10	Age *F* = 30.240; genotype *F* = 48.714; age × genotype *F* = 2.404	Age *p* < 0.001; genotype *p* < 0.001; age × genotype *p* = 0.100	Age α = 1.000; genotype α = 1.000; age × genotype α = 0.278
5*B*	Two-way ANOVA	WT: P1 = 5, P6 = 8, P14 = 15; KO: P1 = 5, P6 = 10, P14 = 16	Age *F* = 74.602; genotype *F* = 1.300; age × genotype *F* = 0.839	Age *p* < 0.001; genotype *p* = 0.259; age × genotype *p* = 0.438	Age α = 1.000; genotype α = 0.0789; age × genotype α = 0.050
5*C*	Two-way ANOVA	WT: P1 = 5, P6 = 8, P14 = 15; KO: P1 = 5, P6 = 10, P14 = 16	Age *F* = 6.544; genotype *F* = 0.049; age × genotype *F* = 0.730	Age *p* = 0.003; genotype *p* = 0.824; age × genotype *p* = 0.487	Age α = 0.844; genotype α = 0.050; age × genotype α = 0.050
5*E*	Two-way ANOVA	WT: P1 = 5, P6 = 8, P14 = 15; KO: P1 = 5, P6 = 10, P14 = 16	Age *F* = 188.708; genotype *F* = 1.683; age × genotype *F* = 0.860	Age *p* < 0.001; genotype *p* = 0.200; age × genotype *p* = 0.428	Age α = 1.000; genotype α = 0.120; age × genotype α = 0.050
5*F*	Two-way ANOVA	WT: P1 = 5, P6 = 8, P14 = 15; KO: P1 = 5, P6 = 10, P14 = 16	Age *F* = 28.024; genotype *F* = 0.009; age × genotype *F* = 0.473	Age *p* < 0.001; genotype *p* = 0.925; age × genotype *p* = 0.626	Age α = 1.000; genotype α = 0.050; age × genotype α = 0.050
5*H*	Two-way ANOVA	WT: P1 = 5, P6 = 8, P14 = 15; KO: P1 = 5, P6 = 10, P14 = 16	Age *F* = 60.344; genotype *F* = 0.792; age × genotype *F* = 0.155	Age *p* < 0.001; genotype *p* = 0.377; age × genotype *p* = 0.857	Age α = 1.000; genotype α = 0.050; age × genotype α = 0.050
5*I*	Two-way ANOVA	WT: P1 = 5, P6 = 8, P14 = 15; KO: P1 = 5, P6 = 10, P14 = 16	Age *F* = 35.620; genotype *F* = 0.724; age × genotype *F* = 0.620	Age *p* < 0.001; genotype *p* = 0.398; age × genotype *p* = 0.541	Age α = 1.000; genotype α = 0.050; age × genotype α = 0.050
6*D*	Two-way ANOVA	WT: P6 = 7, P14 = 10; KO:P6 = 13, P14 = 16	Age *F* = 30.666; genotype *F* = 0.009; age × genotype *F* = 0.002	Age *p* < 0.001; genotype *p* = 0.926; age × genotype *p* = 0.967	Age α = 1.000; genotype α = 0.050; age × genotype α = 0.050
6*E*	Two-way ANOVA	WT: P6 = 7, P14 = 10; KO: P6 = 13, P14 = 16	Age *F* = 10.846; genotype *F* = 0.098; age × genotype *F* = 0.078	Age *p* = 0.002; genotype *p* = 0.756; age × genotype *p* = 0.782	Age α = 0.883; genotype α = 0.050; age × genotype α = 0.050
6*F*	Two-way ANOVA	WT: P6 = 7, P14 = 10; KO: P6 = 13, P14 = 16	Age *F* = 1.350; genotype *F* = 3.178; age × genotype *F* = 0.008	Age *p* = 0.255; genotype *p* = 0.085; age × genotype *p* = 0.927	Age α = 0.083; genotype α = 0.282; age × genotype α = 0.050
6*G*	Two-way ANOVA	WT: P6 = 7, P14 = 10; KO: P6 = 13, P14 = 16	Age *F* = 15.330; genotype *F* = 0.195; age × genotype *F* = 0.094	Age *p* < 0.001; genotype *p* = 0.661; age × genotype *p* = 0.760	Age α = 0.972; genotype α = 0.050; age × genotype α = 0.050
7*D*	Two-way ANOVA	WT: P6 = 8, P14 = 10; KO: P6 = 13, P14 = 16	Age *F* = 0.283; genotype *F* = 0.417; age × genotype *F* = 1.336	Age *p* = 0.600; genotype *p* = 0.525; age × genotype *p* = 0.260	Age α = 0.050; genotype α = 0.050; age × genotype α = 0.081
7*E*	Two-way ANOVA	WT: P6 = 8, P14 = 10; KO: P6 = 13, P14 = 16	Age *F* = 0.804; genotype *F* = 66.730; age × genotype *F* = 0.017	Age *p* = 0.379; genotype *p* < 0.001; age × genotype *p* = 0.896	Age α = 0.050; genotype α = 1.000; age × genotype α = 0.050
7*F*	Two-way ANOVA	WT: P6 = 8, P14 = 10; KO:P6 = 13, P14 = 16	Age *F* = 1.785; genotype *F* = 4.347; age × genotype *F* = 0.037	Age *p* = 0.194; genotype *p* = 0.047; age × genotype *p* = 0.849	Age α = 0.128; genotype α = 0.408; age × genotype α = 0.050
7*G*	Two-way ANOVA	WT: P6 = 8, P14 = 10; KO: P6 = 13, P14 = 16	Age *F* = 1.291; genotype *F* = 23.979; age × genotype *F* = 0.719	Age *p* = 0.268; genotype *p* < 0.001; age × genotype *p* = 0.406	Age α = 0.077; genotype α = 0.998; age × genotype α = 0.050
8*D*	Two-way ANOVA	WT: P6 = 8, P14 = 10; KO: P6 = 13, P14 = 16	Age *F* = 16.857; genotype *F* = 0.210; age × genotype *F* = 0.169	Age *p* < 0.001; genotype *p* = 0.649; age × genotype *p* = 0.683	Age α = 0.984; genotype α = 0.050; age × genotype α = 0.050
8*E*	Two-way ANOVA	WT: P6 = 8, P14 = 10; KO: P6 = 13, P14 = 16	Age *F* = 13.242; genotype *F* = 0.991; age × genotype *F* = 0.078	Age *p* < 0.001; genotype *p* = 0.325; age × genotype *p* = 0.782	Age α = 0.944; genotype α = 0.050; age × genotype α = 0.050
8*F*	Two-way ANOVA	WT: P6 = 8, P14 = 10; KO: P6 = 13, P14 = 16	Age *F* = 5.388; genotype *F* = 40.622; age × genotype *F* = 0.008	Age *p* < 0.001; genotype *p* = 0.027; age × genotype *p* = 0.929	Age α = 1.000; genotype α = 0.519; age × genotype α = 0.050
8*G*	Two-way ANOVA	WT: P6 = 8, P14 = 10; KO: P6 = 13, P14 = 16	Age *F* = 62.677; genotype *F* = 4.302; age × genotype *F* = 1.233	Age *p* < 0.001; genotype *p* = 0.044; age × genotype *p* = 0.273	Age α = 1.000; genotype α = 0.414; age × genotype α = 0.072
9*D*	Two-way ANOVA	WT: P1 = 7, P6 = 8, P14 = 15; KO: P1 = 5, P6 = 14, P14 = 16	Age *F* = 24.955; genotype *F* = 1.250; age × genotype *F* = 0.125	Age *p* < 0.001; genotype *p* = 0.269; age × genotype *p* = 0.882	Age α = 1.000; genotype α = 0.074; age × genotype α = 0.050
9*H*	Two-way ANOVA	WT: P1 = 7, P6 = 8, P14 = 15; KO: P1 = 5, P6 = 14, P14 = 16	Age *F* = 52.928; genotype *F* = 0.219; age × genotype *F* = 0.428	Age *p* < 0.001; genotype *p* = 0.642; age × genotype *p* = 0.654	Age α = 1.000; genotype α = 0.050; age × genotype α = 0.050
9*L*	Two-way ANOVA	WT: P1 = 7, P6 = 8, P14 = 15; KO: P1 = 5, P6 = 14, P14 = 16	Age *F* = 50.641; genotype *F* = 0.783; age × genotype *F* = 1.917	Age *p* < 0.001; genotype *p* = 0.380; age × genotype *p* = 0.156	Age α = 1.000; genotype α = 0.050; age × genotype α = 0.193
10*B*	Two-way ANOVA	WT: P6 = 8, P14 = 13; KO: P6 = 10, P14 = 11	Age *F* = 21.766; genotype *F* = 5.393; age × genotype *F* = 1.687	Age *p* < 0.001; genotype *p* = 0.026; age × genotype *p* = 0.203	Age α = 0.997; genotype α = 0.524; age × genotype α = 0.119
10*D*	Two-way ANOVA	WT: P6 = 8, P14 = 13; KO: P6 = 10, P14 = 11	Age *F* = 19.654; genotype *F* = 1.462; age × genotype *F* = 0.256	Age *p* < 0.001; genotype *p* = 0.234; age × genotype *p* = 0.616	Age α = 0.994; genotype α = 0.095; age × genotype α = 0.050
10*F*	Two-way ANOVA	WT: P6 = 8, P14 = 13; KO: P6 = 10, P14 = 11	Age *F* = 27.231; genotype *F* = 5.368; age × genotype *F* = 2.468	Age *p* < 0.001; genotype *p* = 0.026; age × genotype *p* = 0.125	Age α = 1.000; genotype α = 0.523; age × genotype α = 0.205

## Results

To evaluate developmental differences in the *Fmr1* KO auditory brainstem, we studied wild-type and *Fmr1* KO mice at P1, P6, and P14. P1 represents an age when VCN, MNTB, and LSO are present, but the inputs to each nucleus are not yet fully formed (Morest, 1968; Hoffpauir et al., 2006). At P6, an age before hearing onset, synaptic inputs are present in these nuclei and have begun to mature, and spontaneous activity is evident in the auditory brainstem (Hoffpauir et al., 2010; Holcomb et al., 2013; Wang and Bergles, 2015). By P14, just after hearing onset, inputs to nuclei have undergone considerable pruning (Hoffpauir et al., 2006). Here, we analyze the genesis of the cellular and synaptic abnormalities found in adult *Fmr1* KO mice to investigate the emergence of adult *Fmr1* KO auditory brainstem phenotypes.

### Cell size is significantly reduced in Fmr1 KO auditory brainstem nuclei

Previous studies have shown that cell sizes in the auditory brainstem nuclei of adult *Fmr1* KO animals were significantly reduced in VCN and in MNTB, but not in LSO (Rotschafer et al., 2015; Ruby et al., 2015). Here, we investigated the developmental emergence of this phenotype. We compared cell size by genotype and age within VCN, MNTB, and LSO. As MNTB is known to have a medial-lateral cell size gradient (Pasic and Rubel, 1991; Weatherstone et al., 2017), we also analyzed the medial and lateral portions of MNTB separately then assessed the development of the gradient by comparing the size of cells in the medial to those in the lateral MNTB.

#### VCN

**Figure 1.**
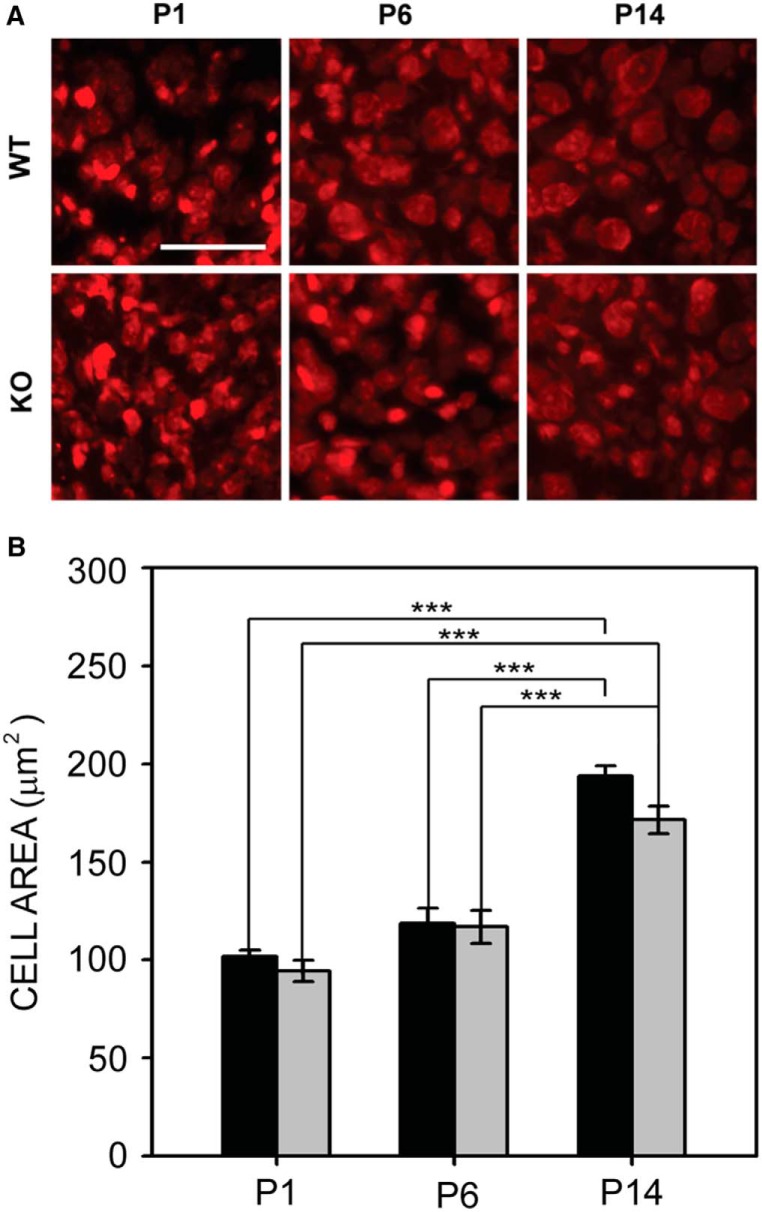
VCN cell size in wild-type and *Fmr1* KO mice. ***A***, VCN cells stained with fluorescent Nissl in wild-type (top) and *Fmr1* KO (bottom) mice at P1, P6, and P14. Scale bar = 100 μm. ***B***, Cell size did not differ between wild-type mice (black bars) and *Fmr1* KO mice (gray bars) at P1, P6, or P14. Both genotypes showed increases in cell size between P1 and P14, and between P6 and P14. *, *p* < 0.05; **, *p* < 0.01; and ***, *p* < 0.001.

We examined VCN cell size in fluorescent Nissl sections at P1, P6, and P14 (Fig. 1*A*). We did not observe a significant difference in cell size between wild-type and *Fmr1* KO at P1, P6, or P14 (*F*_1,60_ = 2.714, *p* = 0.105; Fig. 1*B*), although cells got larger with age in both genotypes (*F*_2,60_ = 74.469, *p* < 0.001). No interaction was seen between the effects of genotype and age. The mean cell size for wild-type mice was 101.9 ± 3.0 μm^2^ at P1, 118.9 ± 7.4 μm^2^ at P6, and 193.8 ± 5.1 μm^2^ at P14. In *Fmr1* KO mice, the mean cell size was 94.4 ± 5.3 μm^2^ at P1, 116.9 ± 8.3 μm^2^ at P6, and 171.5 ± 7.0 μm^2^ at P14 (Fig. 1*A*). These data suggest that the adult-like phenotype of reduced VCN cell size is evident only after hearing onset.

#### MNTB

**Figure 2.**
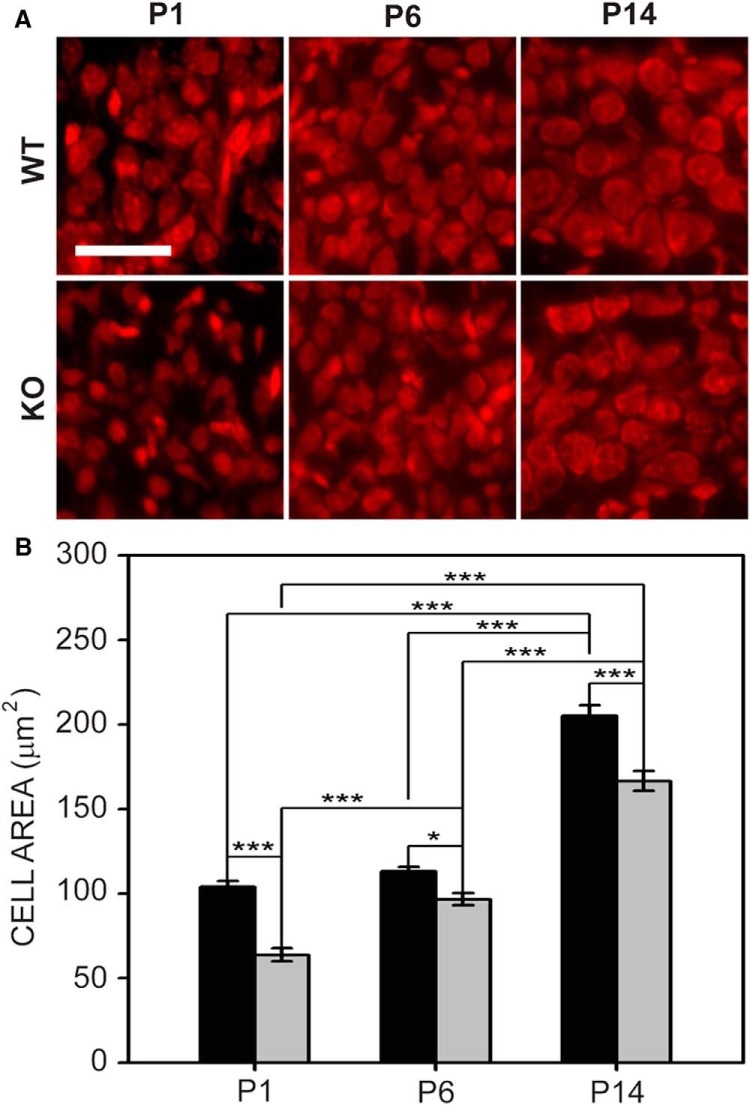
MNTB cell size in wild-type and *Fmr1* KO mice. ***A***, MNTB cells in wild-type mice (top) and *Fmr1* KO mice (bottom) at P1, P6, and P14. Scale bar = 100 μm. ***B***, The cross-sectional area of *Fmr1* KO neurons was significantly reduced compared to wild-type mice at all ages tested. *, *p* < 0.05; **, *p* < 0.01; and ***, *p* < 0.001.

*Fmr1* KO MNTB cells were significantly smaller than wild-type MNTB cells at all ages tested (Fig. 2*A*, *B*). Both wild-type and *Fmr1* KO MNTB cells significantly increased in size with age (*F*_2,43_ = 234.948, *p* < 0.001). Cells were significantly smaller in *Fmr1* KO MNTB (*F*_2,43_ = 56.277, *p* < 0.001). The mean cross-sectional area of wild-type MNTB cells was 108.2 ± 3.5 μm^2^ at P1, 113.2 ± 2.6 μm^2^ at P6, and 204.9 ± 6.3 μm^2^ at P14. In contrast, the mean cross-sectional area of MNTB cells in *Fmr1* KO mice was 63.9 ± 3.9 μm^2^ at P1, 96.8 ± 3.6 μm^2^ at P6, and 166.5 ± 5.9 μm^2^ at P14. No significant interactions were seen between age and genotype.

**Figure 3.**
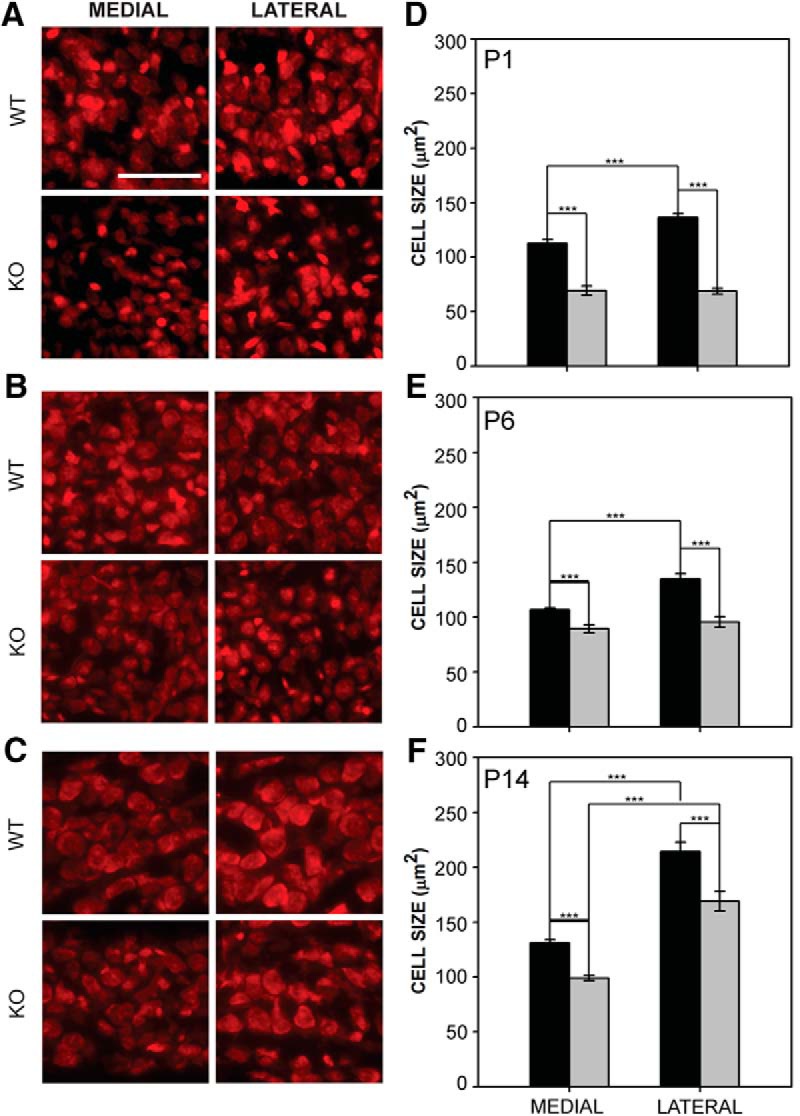
MNTB cell size was reduced and medial-lateral cell size gradient development delayed in *Fmr1* KO mice. ***A–C***, Cell area in wild-type and *Fmr1* KO MNTB at P1, P6, and P14. ***D–F***, Cells in the *Fmr1* KO medial and lateral MNTB were smaller than those found in wild-type mice at P1, P6, and P14. In P1 and P6 wild-type mice, medial MNTB cells were significantly smaller than lateral MNTB cells, but in *Fmr1* KO mice, this difference was not observed. By P14, both genotypes showed significantly smaller cell size in medial MNTB compared to lateral MNTB. Scale bar in ***A*** = 100 μm; applies to ***A–C***. *, *p* < 0.05; **, *p* < 0.01; and ***, *p* < 0.001.

Because FMRP is expressed along a medial-lateral gradient in MNTB (Ruby et al., 2015) we tested whether FMRP deletion impairs the development of the cell size gradient found in MNTB (Weatherstone et al., 2017). We examined cell size in medial and lateral portions of wild-type and *Fmr1* KO mice in MNTB at P1 (Fig. 3*A*), P6 (Fig. 3*B*), and P14 (Fig. 3*C*). We found that cell size at P1 was significantly different between genotypes (*F*_1,20_ = 240.432, *p* < 0.001) and locations (*F*_1,20_ = 10.762, *p* = 0.004; Fig. 3*D*). Although cells in the medial portion of the MNTB in wild-type mice were significantly smaller than those in the lateral portion, there was no medial versus lateral difference in *Fmr1* KO mice (Figs. 3*D*) at this age. Similarly, at P6 (Fig. 3*E*) cell size was significantly different between genotypes (*F*_1,40_ = 39.594, *p* < 0.001) and locations (*F*_1,40_ = 14.142, *p* < 0.001). Medial and lateral MNTB cell sizes were significantly different in wild-type mice, but not different in *Fmr1* KO mice. An interaction between location and genotype was seen at P1 and P6. At P14 (Fig. 3*F*), cell size by both genotype (*F*_1,58_ = 36.820, *p* < 0.001) and cell location (F_1,58_ = 144.012, *p* < 0.001) was significantly different, but no interaction was observed at this age. In the medial portion of MNTB, wild-type cells were 112.6 ± 3.5 μm^2^ at P1, 106.8 ± 1.5 μm^2^ at P6, and 131.1 ± 2.7 μm^2^ at P14. In *Fmr1* KO medial MNTB, cells were 69.3 ± 4.4 μm^2^ at P1, 89.5 ± 3.6 μm^2^ at P6, and 98.9 ± 2.3 μm^2^ at P14. In wild-type mice, lateral MNTB cells were 136.5 ± 3.3 μm^2^ at P1, 134.6 ± 5.1 μm^2^ at P6, and 214.3 ± 8.4 μm^2^ at P14. In *Fmr1* KO mice, lateral MNTB cells were 68.8 ± 2.6 μm^2^ at P1, 95.5 ± 4.9 μm^2^ at P6, and 169.0 ± 8.9 μm^2^ at P14. Thus, while MNTB cells were smaller in *Fmr1* KO mice throughout the nucleus at all ages tested, we found that a gradient of cell size emerged early in wild-type mice and was evident by P14 in both *Fmr1* KO and wild-type mice.

#### LSO

**Figure 4.**
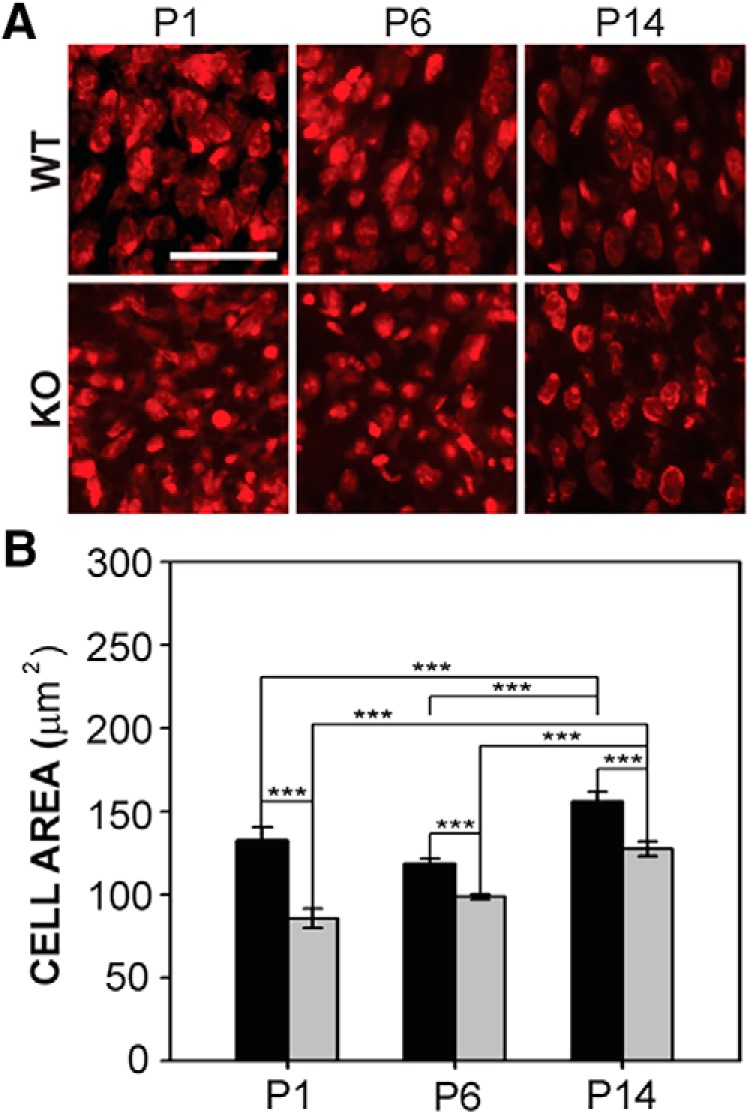
LSO cell size in wild-type and *Fmr1* KO mice. ***A***, Fluorescent Nissl stain in LSO in wild-type mice (top) and *Fmr1* KO mice (bottom) at P1, P6, and P14. Scale bar = 100 μm. ***B***, *Fmr1* KO cell size was significantly smaller than those in wild-type mice at all ages tested. *, *p* < 0.05; **, *p* < 0.01; and ***, *p* < 0.001.

We found that *Fmr1* deletion was associated with reduced cell size in LSO (Fig. 4*A*). In wild-type mice, the mean cross-sectional area of LSO cells was 132.4 ± 8.0 μm^2^ at P1, 118.7 ± 3.1 μm^2^ at P6, and 156.0 ± 5.7 μm^2^ at P14. In *Fmr1* KO mice, LSO cells measured 85.9 ± 5.9 μm^2^ at P1, 98.8 ± 1.5 μm^2^ at P6, and 127.4 ± 4.4 μm^2^ at P14. Cell size in *Fmr1* KO mice was significantly smaller than that seen in wild-type mice at P1, P6, and P14 (*F*_1,56_ = 48.714, *p* < 0.001; Fig. 4*B*). Significant differences in LSO cell size were also found with age (*F*_2,56_ = 30.240, *p* < 0.001) for wild-type and *Fmr1* KO mice (Fig. 4*B*). Cell size increased at each age in *Fmr1* KO mice. Thus, whereas adult *Fmr1* KO mice show normal cell size in LSO, a developmental delay in the growth of these cells is observed in these mutants.

### Correlation of cell size across nuclei

Because cell size reduction was a prominent phenotype in all three nuclei, we tested whether cell size was correlated between nuclei in individual animals. We obtained Pearson correlations among the three nuclei separately for mutant and wild-type animals and for both ages. After Bonferroni correction, we did not find significant correlations at for either genotype at P6. We found that cell size in VCN was positively correlated with cell size in LSO in P14 *Fmr1* KO mice (*p* < 0.002), but the correlation between VCN and LSO in P14 wild-type mice did not reach significance after correction. The statistics are shown in Table 2.

**Table 2.** Correlation coefficients and statistics comparing cell size across nuclei

Source	MNTB cell size	LSO cell size
P6 wild type		
VCN cell size	Pearson’s *r* = 0.014; *p* = 0.975; *n* = 8	Pearson’s *r* = 0.393; *p* = 0.335; *n* = 8
MNTB cell size	—	Pearson’s *r* = –0.375; *p* = 0.360; *n* = 8
P6 Fmr1 KO		
VCN cell size	Pearson’s *r* = 0.675; *p* = 0.096; *n* = 7	Pearson’s *r* = –0.544; *p* = 0.068; *n* = 12
MNTB cell size	—	Pearson’s *r* = –0.785; *p* = 0.037; *n* = 7
P14 wild type		
VCN cell size	Pearson’s *r* = –0.239; *p* = 0.507; *n* = 10	Pearson’s *r* = 0.655; *p* = 0.008; *n* = 15
MNTB cell size	—	Pearson’s *r* = 0.440; *p* = 0.203; *n* = 10
P14 Fmr1 KO		
VCN cell size	Pearson’s *r* = –0.176; *p* = 0.650; *n* = 9	Pearson’s *r* = 0.772; *p* = 0.0012; *n* = 14
MNTB cell size	—	Pearson’s *r* = 0.048; *p* = 0.902; *n* = 9

### Similar auditory nucleus size and number of cells in wild-type and Fmr1 KO mice

#### VCN

**Figure 5.**
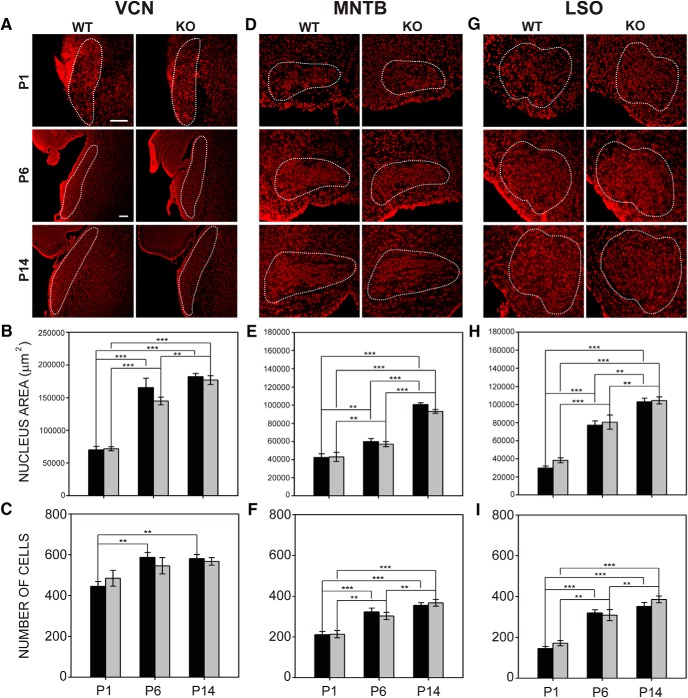
Nucleus growth and cell acquisition in wild-type and *Fmr1* KO mice. ***A***, Outlines of VCN in coronal sections at P1, P6, and P14. Medial is to the right. ***B***, In VCN, both wild-type mice and *Fmr1* KO mice show age-related increases in nucleus area. ***C***, The number of cells in the wild-type VCN increased with age. Increases in cell number in *Fmr1* KO mice did not reach significance. ***D***, Outlines of MNTB in wild-type and *Fmr1* KO mice at P1, P6, and P14. ***E***, In MNTB, wild-type and *Fmr1* KO mice both showed significant increases in nucleus size at all ages tested. ***F***, Both wild-type mice and *Fmr1* KO mice showed age-dependent increases in cell number in MNTB. ***G***, Outlines of LSO in wild-type and *Fmr1* KO mice at P1, P6, and P14. ***H***, LSO grew in size at each age in both wild-type and *Fmr1* KO mice. ***I***, The number of cells in LSO in both wild-type mice and *Fmr1* KO mice increased significantly with age. Top row in ***A***: 100-μm scale bar applies to P1 images (20× magnification). Second row in ***A***: 100-μm scale bar applies to for P6 and P14 images (10× magnification). *, *p* < 0.05; **, *p* < 0.01; and ***, *p* < 0.001.

A two-way ANOVA showed that age (P1, P6, or P14) had a significant effect on nucleus size (*F*_2,53_ = 74.602, *p* < 0.001), but genotype (wild-type or *Fmr1* KO) did not (*F*_1,53_ = 1.300, *p* = 0.259). Pairwise analysis showed that VCN grew at a consistent rate in both wild-type and *Fmr1* KO mice (Fig. 5*A*, *B*). In wild-type mice, the mean area of VCN sections was 70,136 ± 5327 μm^2^ at P1, 165,628 ± 14,272 μm^2^ at P6, and 182,174 ± 5247 μm^2^ at P14. In *Fmr1* KO mice, the mean area of VCN was 71,729 ± 3160 μm^2^ at P1, 144,924 ± 5928 μm^2^ at P6, and 177,055 ± 6652 μm^2^ at P14. As in wild-type mice, the number of cells in VCN at different ages increased significantly (*F*_2,53_ = 6.544, *p* = 0.003). Wild-type mice gained cells in VCN from P1 (445.6 ± 23.2 cells) to P6 (587.2 ± 24.4 cells), but not from P6 to P14 (581.1 ± 19.7 cells). VCN in *Fmr1* KO mice also showed a significant gain in cell numbers between P1 (484.8 ± 38.9 cells) and P6 (545.4 ± 40.1), and between P6 and P14 (567.2 ± 18.2 cells). Both *Fmr1* KO and wild-type mice showed a significant increase in cell number between P1 and P14 (Fig. 5*C*).

#### MNTB

Analysis of MNTB also showed significant differences in nucleus size with age (*F*_2,59_ = 188.708, *p* < 0.001), but not with genotype (*F*_1,59_ = 1.683, *p* = 0.200; Fig. 5*D*). In wild-type mice, there was a significant increase in MNTB volume between P1 (42,502 ± 3856 μm^2^) and P6 (59,974 ± 3310 μm^2^), and between P6 and P14 (100,662 ± 1976 μm^2^). MNTB size in *Fmr1* KO mice also showed gains at each age tested; a significant change in MNTB size was found between P1 (42,979 ± 5051 μm^2^) and P6 (57,020 ± 2837 μm^2^), and a significant increase was seen between P6 and P14 (93,280 ± 2108 μm^2^; Fig. 5*E*). The number of cells within MNTB increased significantly with age (*F*_2, 59_ = 28.024, *p* < 0.001), though the number of cells did not differ by genotype (F_1,59_ = 0.009, *p* = 0.925). In both wild-type and *Fmr1* KO mice, the number of cells in MNTB increased significantly from P1 to P6, but the increase from P6 to P14 did not reach significance in wild-type mice (Fig. 5*F*). In wild-type mice, there were 210.5 ± 16.5 cells in MNTB sections at P1, 322.7 ± 18.8 cells at P6, and 355.2 ± 12.7 cells at P14. *Fmr1* KO mice had 213.8 ± 18.2 cells in MNTB at P1, 302.8 ± 18.1 cells at P6, and 367.3 ± 16.8 cells at P14. The growth of MNTB thus does not appear to differ between mutants and wild-type mice.

#### LSO

Similar to VCN and MNTB findings, LSO nucleus size and number of cells did not differ between wild-type and *Fmr1* KO mice. LSO mean cross-sectional area significantly increased with age in both wild-type and *Fmr1* KO mice (*F*_2,59_ = 60.344, *p* < 0.001), but did not vary by genotype (*F*_1,59_ = 0.792, *p* = 0.377; Fig. 5*G*). LSO size increased significantly from P1 to P6 and from P1 to P14 in wild-type and *Fmr1* KO mice, and *Fmr1* KO LSO also showed an increase from P6 to P14 (Fig. 5*H*). At P1, the LSO was 29,692 ± 2257 μm^2^ in wild-type mice and 38,352 ± 2725 μm^2^ in *Fmr1* KO mice. At P6, the wild-type LSO was 77,281 ± 4659 μm^2^ and the *Fmr1* KO LSO was 80,564 ± 7872 μm^2^. At P14, LSO in wild-type mice was 102,910 ± 4226 μm^2^ and 104,473 ± 3938 μm^2^ in *Fmr1* KO mice. The number of cells within LSO also increased with age (*F*_2, 59_ = 35.620, *p* < 0.001), but not with genotype (*F*_1,59_ = 0.724, *p* = 0.398; Fig. 5*I*). Both wild-type and *Fmr1* KO mice showed significant increases in the number of LSO cells from P1 (WT: 144.8 ± 10.7; KO: 171.5 ± 12.5) to P6 (WT: 319.8 ± 15.3; KO: 309.0 ± 27.6) and from P1 to P14 (WT: 351.4 ± 19.7; KO: 385.9 ± 17.1). This finding is consistent with data showing no significant difference in the LSO cell density of adult *Fmr1* KO mice (Rotschafer et al., 2015).

### Inhibitory and excitatory synaptic markers in the developing auditory brainstem

Levels of VGLUT and VGAT, markers for excitatory and inhibitory synaptic inputs, respectively, have been shown to be altered in the auditory brainstem in adult *Fmr1* KO mice (Rotschafer et al., 2015; Garcia-Pino et al., 2017). We thus examined the developmental emergence of changes in markers of synaptic input in wild-type and mutant mice.

#### VCN

**Figure 6.**
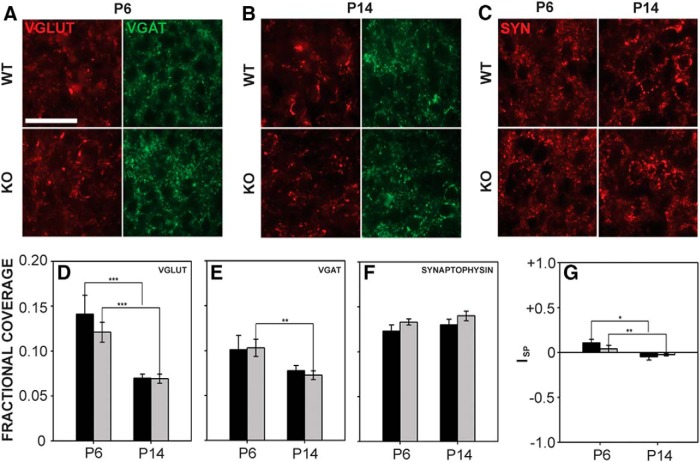
VGLUT, VGAT, and synaptophysin expression in VCN. ***A***, ***B***, VGLUT and VGAT immunofluorescence in VCN in wild-type (top) and *Fmr1* KO (bottom) mice at P6 (***A***) and P14 (***B***). ***C***, Synaptophysin expression in VCN in wild-type (top) and *Fmr1* KO (bottom) mice at P6 (left) and P14 (right). ***D***, In VCN in both wild-type and *Fmr1* KO mice, VGLUT fractional coverage was reduced at P14 compared to P6, but did not vary between genotypes. ***E***, VGAT fractional coverage decreased significantly from P6 to P14 in *Fmr1* KO mice. ***F***, Synaptophysin fractional coverage did not differ with age or genotype. ***G***, VGLUT relative to VGAT expression (I_SP_) did not differ significantly between genotypes, but I_SP_ values decreased significantly from P6 to P14. Scale bar in ***A*** = 50 μm; applies to ***A–C***. *, *p* < 0.05; **, *p* < 0.01; and ***, *p* < 0.001.

The expression values of VGLUT and VGAT in VCN are shown in Fig. 6*A*, *B*. In addition, we examined expression of synaptophysin during development to evaluate the numbers of presynaptic inputs (Fig. 6*C*). In VCN, the fractional coverage of VGLUT and VGAT significantly decreased with age (VGLUT: *F*_1,43_ = 30.666, *p* < 0.001; VGAT: *F*_1,43_ = 10.846, *p* = 0.002), but no difference in the amount of VGLUT or VGAT was found between genotypes at any age tested (VGLUT: *F*_1,43_ = 0.009, *p* = 0.926; VGAT: *F*_1,43_ = 0.098, *p* = 0.756; Fig. 6*D*, *E*). VGLUT fractional coverage in the wild-type VCN was 0.14 ± 0.02 at P6 and 0.07 ± 0.004 at P14, whereas *Fmr1* KO fractional coverage was 0.12 ± 0.01 at P6 and 0.07 ± 0.005 at P14. VGAT fractional coverage in wild-type mice was 0.10 ± 0.02 at P6 and 0.08 ± 0.01 at P14. In *Fmr1* KO mice, VGAT fractional coverage was 0.10 ± 0.01 at P6 and 0.07 ± 0.004 at P14. Using synaptophysin fractional coverage, we determined that there was no significant difference in the amount of synaptic inputs to VCN between genotypes (*F*_1,29_ = 3.178, *p* = 0.085) or ages (*F*_1,29_ = 1.350, *p* = 0.255; Fig. 6*F*). Wild-type synaptophysin fractional coverage at P6 was 0.12 ± 0.007 and at P14 was 0.13 ± 0.006; *Fmr1* KO coverage at P6 was 0.13 ± 0.003 and at P14 was 0.14 ± 0.005. The relative levels of VGLUT and VGAT, expressed as I_SP_ values, were significantly different for age (*F*_1,43_ = 15.330, *p* < 0.001; Fig. 6*G*) but not for genotype (*F*_1,43_ = 0.195, *p* = 0.661). At P6, the I_SP_ value was 0.12 ± 0.04 in wild-type mice and 0.04 ± 0.04 in *Fmr1* KO mice. At P14, the I_SP_ value was –0.05 ± 0.04 in wild-type mice and –0.03 ± 0.01 in *Fmr1* KO mice. Thus, as in adult *Fmr1* KO mice, synaptic inputs to VCN were not altered.

#### MNTB

**Figure 7.**
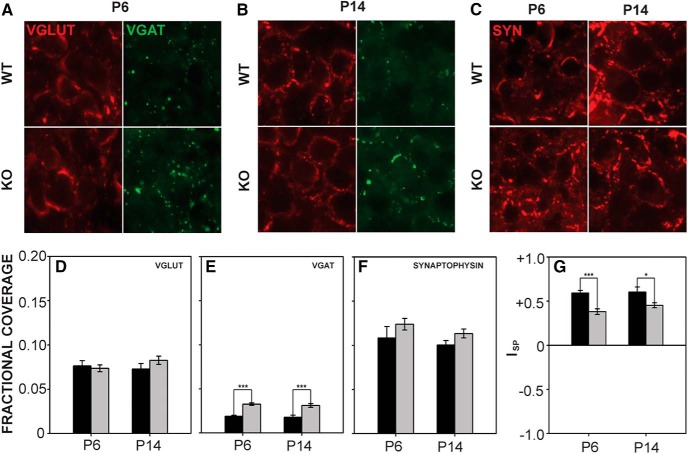
VGLUT, VGAT, and synaptophysin expression in MNTB. ***A***, ***B***, VGLUT and VGAT expression in wild-type and *Fmr1* KO MNTB at P6 (***A***) and P14 (***B***). ***C***, Synaptophysin expression in MNTB in wild-type (top) and *Fmr1* KO (bottom) mice. ***D***, VGLUT fractional coverage did not vary between genotypes or ages. ***E***, VGAT fractional coverage was significantly greater in *Fmr1* KO MNTB at both P6 and P14. ***F***, Synaptophysin coverage did not differ significantly by age or by genotype. ***G***, I_SP_ values were significantly smaller in *Fmr1* KO mice at both P6 and P14. Scale bar in ***A*** = 50 μm; applies to ***A–C***. *, *p* < 0.05; **, *p* < 0.01; and ***, *p* < 0.001.

We evaluated expression of presynaptic markers in MNTB (Fig. 7*A–C*). VGLUT fractional coverage was similar between the genotypes (*F*_1,22_ = 0.417, *p* = 0.525; Fig. 7*D*), but VGAT fractional coverage was significantly greater in *Fmr1* KO mice (*F*_1,22_ = 66.730, *p* < 0.001; Fig. 7*E*) at both ages. Neither VGLUT nor VGAT coverage changed significantly from P6 to P14 (VGLUT: *F*_1,22_ = 0.283, *p* = 0.600; VGAT: *F*_1,22_ = 0.804, *p* = 0.379). At P6, wild-type VGLUT fractional coverage was 0.08 ± 0.01 and VGAT coverage was 0.02 ± 0.001. P6 *Fmr1* KO fractional coverage for VGLUT was 0.07 ± 0.003 and for VGAT was 0.03 ± 0.001. At P14, VGLUT coverage in wild-type mice was 0.07 ± 0.01 and in *Fmr1* KO mice was 0.08 ± 0.005; VGAT coverage in wild-type mice was 0.02 ± 0.002 and in *Fmr1* KO mice was 0.03 ± 0.002. Overall, synaptophysin fractional coverage was significantly greater in *Fmr1* KO mice than in wild-type mice (*F*_1,25_ = 4.347, *p* = 0.047; Fig. 7*C*, *F*); however, *post hoc* analysis at each age did not reach significance (*F*_1,25_ = 1.785, *p* = 0.194). At P6, synaptophysin coverage in wild-type mice was 0.10 ± 0.01 and 0.12 ± 0.02 in *Fmr1* KO mice. Wild type coverage at P14 was 0.10 ± 0.005, and *Fmr1* KO coverage was 0.12 ± 0.005. Relative levels of VGLUT and VGAT expressed as I_SP_ values (Fig. 7*G*) were significantly smaller in *Fmr1* KO mice than in wild-type mice (*F*_1,22_ = 23.979, *p* < 0.001), indicative of reduced excitatory input relative to inhibitory input. No changes in I_SP_ values were found with age (*F*_1,22_ = 1.291, *p* = 0.268). I_SP_ values in wild-type mice were 0.59 ± 0.03 at P6 and 0.60 ± 0.06 at P14. In *Fmr1* KO mice, I_SP_ values were 0.38 ± 0.03 at P6 and 0.45 ± 0.03 at P14. These results suggest that greater VGAT fractional coverage values drive a decrease in *Fmr1* KO I_SP_ values and that this phenotype is present early in postnatal development.

#### LSO

**Figure 8.**
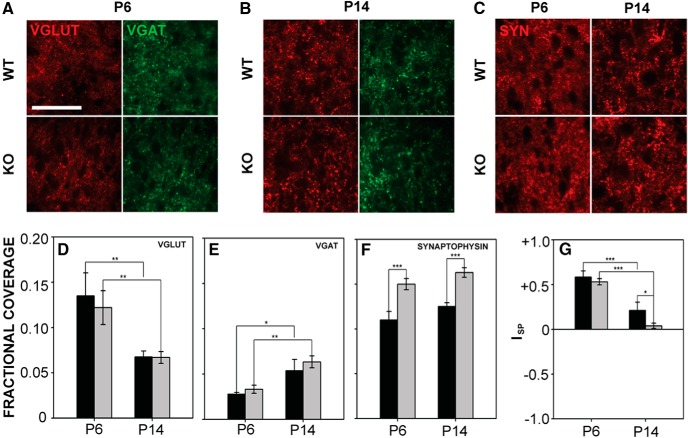
VGLUT, VGAT, and synaptophysin expression in LSO. ***A***, ***B***, Expression of VGLUT (left) and VGAT (right) at P6 and P14 in LSO. ***C***, Synaptophysin expression at P6 and P14 in wild-type and mutant mice. ***D***, In both wild-type and *Fmr1* KO LSO, VGLUT expression decreased with age but did not differ between genotypes. ***E***, VGAT fractional coverage increased between P6 and P14 in both genotypes but did not differ between genotypes. ***F***, Synaptophysin fractional coverage was significantly greater in *Fmr1* KO mice at both ages tested and did not change significantly between these ages. ***G***, I_SP_ values decreased significantly between P6 and P14. I_SP_ was significantly smaller in *Fmr1* KO mice than in wild-type mice at P14. Scale bar in ***A*** = 50 μm; applies to ***A–C***. *, *p* < 0.05; **, *p* < 0.01; and ***, *p* < 0.001.

VGLUT, VGAT, and synaptophysin expression were examined in LSO (Fig. 8*A–C*). VGLUT and fractional coverage significantly decreased with age in wild-type and *Fmr1* KO mice, and VGAT coverage increased with age (VGLUT: *F*_1,43_ = 16.857, *p* < 0.001; VGAT: *F*_1,43_ = 13.242, *p* < 0.001), although there was no difference between genotypes (VGLUT: *F*_1,43_ = 0.210, *p* = 0.649; VGAT: *F*_1,43_ = 0.991, *p* = 0.325; Fig. 8*D*, *E*). Fractional VGLUT coverage was 0.14 ± 0.03 at P6 and 0.07 ± 0.006 at P14 in wild-type mice, and 0.12 ± 0.02 at P6 and 0.07 ± 0.006 at P14 in *Fmr1* KO mice. VGAT fractional coverage in wild-type mice was 0.03 ± 0.002 at P6 and 0.05 ± 0.01 at P14. In *Fmr1* KO mice, VGAT coverage was 0.03 ± 0.004 at P6 and 0.06 ± 0.007 at P14. Synaptophysin fractional coverage was significantly greater in *Fmr1* KO mice (*F*_1,30_ = 40.622, *p* < 0.001) at both P6 and P14 (Fig. 8*F*). There was also a significant difference in synaptophysin expression between ages (*F*_1,30_ = 5.388, *p* = 0.027). In wild-type mice, LSO I_SP_ values were 0.59 ± 0.07 at P6 and 0.21 ± 0.09 at P14. In *Fmr1* KO mice, I_SP_ values were 0.53 ± 0.04 at P6 and 0.04 ± 0.03 at P14. I_SP_ values differed between genotypes at P14 (*F*_1,43_ = 4.302, *p* = 0.044), and decreased with age (*F*_1,43_ = 62.677, *p* < 0.001).

### Emergence of microglia and astrocytes in wild-type and Fmr1 KO mice

To determine whether the *Fmr1* deletion alters glial populations in the auditory brainstem, we next quantified the number of microglia and astrocytes using immunofluorescence for the markers Iba1 and ALDH1L1, respectively.

#### Microglia

**Figure 9.**
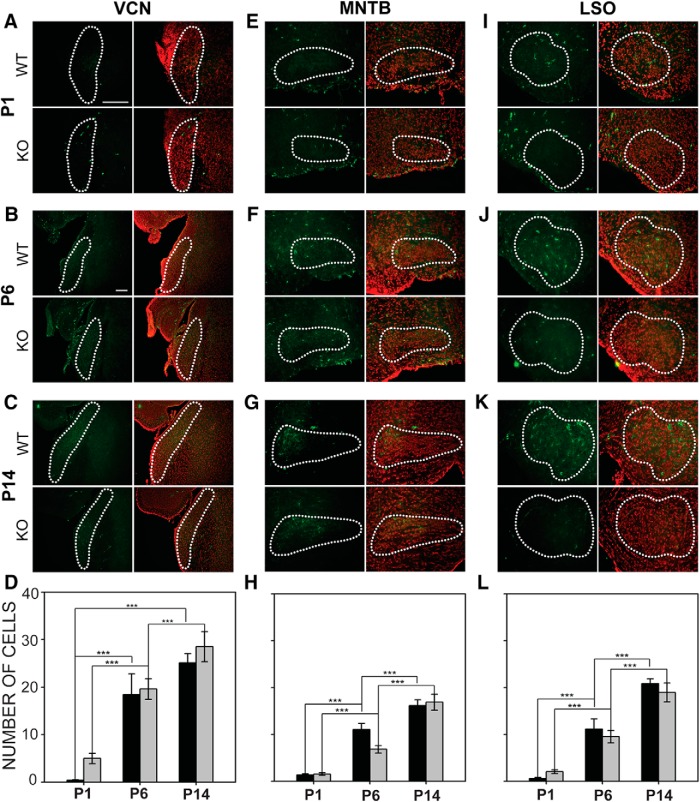
Emergence of microglia in the auditory brainstem nuclei in wild-type and *Fmr1* KO mice. ***A–C***, Increase in microglial population was studied in the VCN at P1, P6, and P14. Left column, Iba1 immunofluorescence in green; right column, Iba1 together with fluorescent Nissl. Nucleus outline is shown in dashed lines. ***D***, The number of microglia in VCN increased with age in both wild-type and *Fmr1* KO mice, but no difference was seen between the genotypes. ***E–G***, Emergence of microglia was evaluated in MNTB using Iba1 immunofluorescence (left) together with Nissl (right). ***H***, Wild type and *Fmr1* KO mice both showed significant increases in the number of microglia present in MNTB each age tested, but no significant differences were found between genotypes. ***I–K***, Emergence of microglia in LSO during development. ***L***, The number of microglia expressed steadily increased at each age tested in both wild-type and *Fmr1* KO mice. As for VCN and MNTB, no difference in numbers of microglia were seen between the genotypes. Scale bar in ***A*** = 100 μm, applies to ***A***, ***E***, and ***I***. Scale bar in ***B*** = 10 0μm, applies to ***B***, ***C***, ***F***, ***G***, and ***J–K***. *, *p* < 0.05; **, *p* < 0.01; and ***, *p* < 0.001.

**Figure 10.**
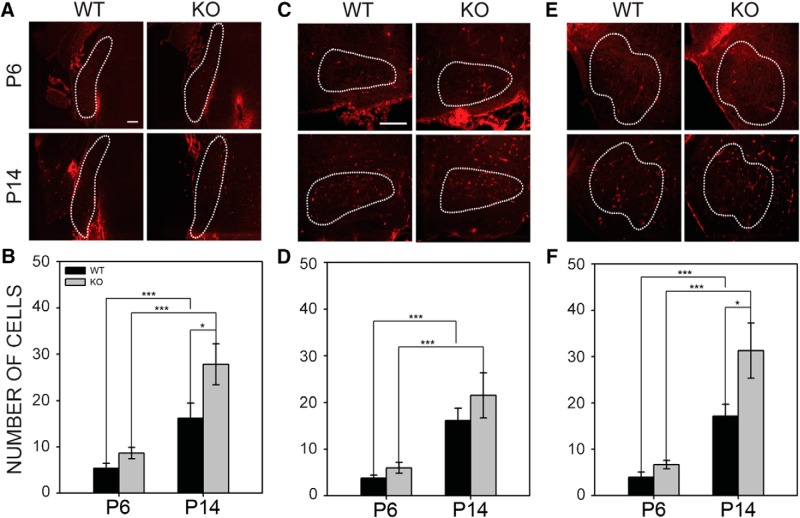
Emergence of astrocytes in the wild-type and *Fmr1* KO auditory brainstem. ***A***, The number of astrocytes in the VCN was evaluated at P6 and P14 using ALDH1L1 immunofluorescence. ***B***, Numbers of ALDH1L1-positive astrocytes in VCN increased between P6 and P14 in both genotypes. At P14, there were significantly more astrocytes in VCN in *Fmr1* KO mice than in wild-type mice. ***C***, Astrocytes labeled in MNTB at P6 and P14. ***D***, Astrocyte numbers increased significantly in MNTB in both wild-type and *Fmr1* KO mice, but no difference were seen between the genotypes. ***E***, ALDH1L1 immunolabeled astrocytes in LSO. ***F***, Astrocytes increased in number between P6 and P14 in both genotypes, and significantly more astrocytes were present in the *Fmr1* KO LSO at P14. Scale bar in ***A*** = 100 μm; scale bar in ***C*** = 100 μm, applies to ***C*** and ***E***. *, *p* < 0.05; **, *p* < 0.01; and ***, *p* < 0.001.

We examined the expression of Iba1 in VCN at P1, P6, and P14 in VCN (Fig. 9*A–C*). A two-way ANOVA revealed that the number of microglia varied with age (*F*_2,53_ = 24.955, *p* <0.001) but not with genotype (*F*_1,53_ = 0.1.250, *p* = 0.269; Fig. 9*D*). Wild-type VCN had an average of 0.4 ± 0.1 microglia per VCN section at P1, 18.43 ± 4.38 at P6, and 25.13 ± 1.94 at P14; *Fmr1* KO VCN had 5.00 ± 1.08 microglia per VCN section at P1, 19.60 ± 2.19 at P6, and 28.52 ± 3.16 at P14. Expression of Iba1 in MNTB (Fig. 9*E–G*) also varied with age (*F*_2,59_ = 52.928, *p* < 0.001; Fig. 10*E–H*) but not with genotype (*F*_1,59_ = 0.219, *p* = 0.642; Fig. 9*H*). Mean numbers of microglia in wild-type MNTB sections were 0.62 ± 0.25 at P1, 11.06 ± 1.30 at P6, and 16.18 ± 1.21 at P14. For *Fmr1* KO mice, mean numbers of microglia were 2.08 ± 0.39 at P1, 6.86 ± 0.78 at P6, and 16.91 ± 1.71 at P14. Similarly, Iba1 expression in LSO (Fig. 9*I–K*) increased with age (*F*_2,59_ = 50.641, *p* < 0.001), but did not depend on genotype (*F*_1,59_ = 0.783, *p* = 0.380; Fig. 9*L*). In wild-type LSO, there was a consistent increase in the number of microglia from P1 (1.37 ± 0.24) to P6 (11.16 ± 2.14), and to P14 (20.88 ± 1.02). In *Fmr1* KO mice, there was a significant difference in the number of microglia in LSO between P1 (1.55 ± 0.29) and P6 (9.53 ± 1.34), between P6 and P14 (18.94 ± 2.01). Thus, no difference between wild-type mice and *Fmr1* KO mice was seen in microglial numbers in these auditory brainstem nuclei. We examined Pearson’s correlation coefficients to determine correlation of the number of microglia with values for VGAT, VGLUT, and synaptophysin in mutants and wild-type animals at P6 and P14 in each nucleus and used Bonferroni correction for these multiple comparisons. A significant positive correlation was found only between Iba1 values and VGLUT values for P6 in MNTB in *Fmr1* KO mice (Table 3).

**Table 3.** Correlations between Iba1 expression levels and expression of each synaptic protein

Comparison, age, and genotype	Nucleus	Correlation coefficient and statistics
Iba1 vs. VGLUT		
P6		
Wild type	VCN	Pearson’s *r* = 0.781; *p* = 0.038; *n* = 7
Wild type	MNTB	Pearson’s *r* = 0.818; *p* = 0.013; *n* = 8
Wild type	LSO	Pearson’s *r* = 0.664; *p* = 0.073; *n* = 8
*Fmr1* KO	VCN	Pearson’s *r* = 0.386; *p* = 0.271; *n* = 10
*Fmr1* KO	MNTB	Pearson’s *r* = 0.793; *p* = 0.001; *n* = 13
*Fmr1* KO	LSO	Pearson’s *r* = 0.305; *p* = 0.310; *n* = 13
P14		
Wild type	VCN	Pearson’s *r* = –0.179; *p* = 0.620; *n* = 10
Wild type	MNTB	Pearson’s *r* = 0.493; *p* = 0.148; *n* = 10
Wild type	LSO	Pearson’s *r* = –0.036; *p* = 0.921; *n* = 10
*Fmr1* KO	VCN	Pearson’s *r* = 0.176; *p* = 0.515; *n* = 16
*Fmr1* KO	MNTB	Pearson’s *r* = –0.039; *p* = 0.887; *n* = 16
*Fmr1* KO	LSO	Pearson’s *r* = –0.392; *p* = 0.133; *n* = 16
Iba1 vs. VGAT		
P6		
Wild type	VCN	Pearson’s *r* = 0.119; *p* = 0.799; *n* = 7
Wild type	MNTB	Pearson’s *r* = 0.737; *p* = 0.037; *n* = 8
Wild type	LSO	Pearson’s *r* = 0.236; *p* = 0.574; *n* = 8
*Fmr1* KO	VCN	Pearson’s *r* = 0.0006; *p* = 0.999; *n* = 10
*Fmr1* KO	MNTB	Pearson’s *r* = 0.695; *p* = 0.008; *n* = 13
*Fmr1* KO	LSO	Pearson’s *r* = 0.130; *p* = 0.673; *n* = 13
P14		
Wild type	VCN	Pearson’s *r* = 0.097; *p* = 0.789; *n* = 10
Wild type	MNTB	Pearson’s *r* = 0.149; *p* = 0.682; *n* = 10
Wild type	LSO	Pearson’s *r* = –0.292; *p* = 0.412; *n* = 10
*Fmr1* KO	VCN	Pearson’s *r* = 0.165; *p* = 0.541; *n* = 16
*Fmr1* KO	MNTB	Pearson’s *r* = –0.345; *p* = 0.190; *n* = 16
*Fmr1* KO	LSO	Pearson’s *r* = –0.401; *p* = 0.124; *n* = 16
Iba1 vs. Synaptophysin		
P6		
Wild type	VCN	Pearson’s *r* = 0.422; *p* = 0.346; *n* = 7
Wild type	MNTB	Pearson’s *r* = 0.006; *p* = 0.989; *n* = 7
Wild type	LSO	Pearson’s *r* = 0.605; *p* = 0.150; *n* = 7
*Fmr1* KO	VCN	Pearson’s *r* = 0.281; *p* = 0.500; *n* = 8
*Fmr1* KO	MNTB	Pearson’s *r* = 0.231; *p* = 0.582; *n* = 8
*Fmr1* KO	LSO	Pearson’s *r* = 0.203; *p* = 0.629; *n* = 8
P14		
Wild type	VCN	Pearson’s *r* = –0.115; *p* = 0.751; *n* = 10
Wild type	MNTB	Pearson’s *r* = 0.346; *p* = 0.328; *n* = 10
Wild type	LSO	Pearson’s *r* = 0.151; *p* = 0.676; *n* = 10
*Fmr1* KO	VCN	Pearson’s *r* = 0.565; *p* = 0.113; *n* = 9
*Fmr1* KO	MNTB	Pearson’s *r* = –0.057; *p* = 0.884; *n* = 9
*Fmr1* KO	LSO	Pearson’s *r* = –0.729; *p* = 0.026; *n* = 9

#### Astrocytes

We next examined the emergence of ALDH1L1-positive astrocytes in the auditory brainstem nuclei at P6 and P14. In VCN (Fig. 10*A*), there was a significant increase in the number of astrocytes with age (*F*_1,35_ = 21.766, *p* < 0.001; Fig. 10*A*, *B*), and in addition, there was a significant difference in the number of astrocytes by genotype at P14 (*F*_1,35_ = 5.393, *p* = 0.026; Fig. 10*B*). Wild-type mice had 5.35 ± 1.10 astrocytes per VCN section at P6 and 16.16 ± 3.28 at P14. *Fmr1* KO mice had 8.63 ± 1.24 at P6 and 27.79 ± 4.45 at P14.

In MNTB, we found that ALDH1L1-positive astrocytes increased with age (*F*_1,38_ = 19.654, *p* < 0.001) but did not differ between genotypes (*F*_1,38_ = 1.462, *p* = 0.234; Fig. 10*C*, *D*). In wild-type mice, the mean number of astrocytes per section was 3.78 ± 0.66 at P6 and 16.11 ± 2.70 at P14. In *Fmr1* KO mice, the mean number of astrocytes per section was 5.99 ± 1.15 at P6, and 21.49 ± 4.80 at P14.

The number of astrocytes present in the LSO was significantly different between the two ages (*F*_1,38_ = 27.231, *p* < 0.001) and also differed between genotypes at P14 (*F*_1,38_ = 5.368, *p* = 0.026). Wild-type and *Fmr1* KO mice gained a significant number of astrocytes between P6 and P14, with significantly more astrocytes present in P14 *Fmr1* KO mice than in wild-type mice. Wild-type mice had 3.95 ± 1.12 astrocytes per section at P6 and 17.17 ± 2.58 at P14; *Fmr1* KO mice had 6.65 ± 0.91 astrocytes per section at P6 and 31.26 ± 5.96 at P14 (Fig. 10*E*, *F*). These results show that *Fmr1* KO mice have significantly more astrocytes in LSO, and that these differences are evident at P14. We did not find any significant correlations between the number of astrocytes and the level of synaptophysin expression in any of the nuclei (Table 4). However, we found a strong positive correlation between ALDH1L1 levels and both VGLUT and VGAT in the P14 LSO in *Fmr1* KO mice. This observation is consistent with astrocyte regulation of synaptic inputs in the mutant LSO (Table 4).

**Table 4.** Correlations between ALDH1L1 expression and synaptic protein expression

Comparison, age, and genotype	Nucleus	Correlation coefficient and statistics
ALDH1L1 vs. VGLUT		
P6		
Wild type	VCN	Pearson’s *r* = 0.056; *p* = 0.906; *n* = 7
Wild type	MNTB	Pearson’s *r* = 0.476; *p* = 0.281; *n* = 7
Wild type	LSO	Pearson’s *r* = 0.141; *p* = 0.763; *n* = 7
*Fmr1* KO	VCN	Pearson’s *r* = 0.248; *p* = 0.489; *n* = 10
*Fmr1* KO	MNTB	Pearson’s *r* = 0.207; *p* = 0.566; *n* = 10
*Fmr1* KO	LSO	Pearson’s *r* = 0.330; *p* = 0.351; *n* = 10
P14		
Wild type	VCN	Pearson’s *r* = 0.450; *p* = 0.225; *n* = 9
Wild type	MNTB	Pearson’s *r* = 0.263; *p* = 0.462; *n* = 10
Wild type	LSO	Pearson’s *r* = –0.238; *p* = 0.508; *n* = 10
*Fmr1* KO	VCN	Pearson’s *r* = –0.507; *p* = 0.135; *n* = 10
*Fmr1* KO	MNTB	Pearson’s *r* = –0.675; *p* = 0.023; *n* = 11
*Fmr1* KO	LSO	Pearson’s *r* = 0.912; *p* = 0.00009; *n* = 11
ALDH1L1 vs. VGAT		
P6		
Wild type	VCN	Pearson’s *r* = –0.775; *p* = 0.041; *n* = 7
Wild type	MNTB	Pearson’s *r* = 0.274; *p* = 0.553; *n* = 7
Wild type	LSO	Pearson’s *r* = 0.057; *p* = 0.903; *n* = 7
*Fmr1* KO	VCN	Pearson’s *r* = 0.028; *p* = 0.939; *n* = 10
*Fmr1* KO	MNTB	Pearson’s *r* = 0.449; *p* = 0.193; *n* = 10
*Fmr1* KO	LSO	Pearson’s *r* = –0.211; *p* = 0.559; *n* = 10
P14		
Wild type	VCN	Pearson’s *r* = 0.407; *p* = 0.277; *n* = 9
Wild type	MNTB	Pearson’s *r* = 0.099; *p* = 0.785; *n* = 10
Wild type	LSO	Pearson’s *r* = –0.436; *p* = 0.208; *n* = 10
*Fmr1* KO	VCN	Pearson’s *r* = –0.721; *p* = 0.019; *n* = 10
*Fmr1* KO	MNTB	Pearson’s *r* = 0.747; *p* = 0.008; *n* = 11
*Fmr1* KO	LSO	Pearson’s *r* = 0.895; *p* = 0.0002; *n* = 11
ALDH1L1 vs. Synaptophysin		
P6		
Wild type	VCN	Pearson’s *r* = –0.434; *p* = 0.331; *n* = 7
Wild type	MNTB	Pearson’s *r* = 0.155; *p* = 0.740; *n* = 7
Wild type	LSO	Pearson’s *r* = 0.550; *p* = 0.201; *n* = 7
*Fmr1* KO	VCN	Pearson’s *r* = 0.376; *p* = 0.359; *n* = 8
*Fmr1* KO	MNTB	Pearson’s *r* = 0.370; *p* = 0.367; *n* = 8
*Fmr1* KO	LSO	Pearson’s *r* = –0.398; *p* = 0.329; *n* = 8
P14		
Wild type	VCN	Pearson’s *r* = –0.126; *p* = 0.747; *n* = 9
Wild type	MNTB	Pearson’s *r* = 0.807; *p* = 0.005; *n* = 10
Wild type	LSO	Pearson’s *r* = 0.136; *p* = 0.707; *n* = 10
*Fmr1* KO	VCN	Pearson’s *r* = –0.077; *p* = 0.857; *n* = 8
*Fmr1* KO	MNTB	Pearson’s *r* = –0.301; *p* = 0.431; *n* = 9
*Fmr1* KO	LSO	Pearson’s *r* = –0.036; *p* = 0.926; *n* = 9

### Auditory brainstem phenotypes in Fmr1 KO mice are not sexually dimorphic

To test for sex differences, we compared our outcome measures by sex in P14 mice. We used two-way ANOVAs to examine whether sex (male or female) within each genotype (wild-type or *Fmr1* KO) caused significant differences in nucleus size, number of cells, cell size, synaptic proteins, or glia number in VCN, MNTB, and LSO. Although any genotype differences found were consistent with previous results, we did not find any significant differences between male and female mice in any measure tested in any nucleus examined (Table 5). We therefore conclude that FMRP loss does not selectively affect cellular or synaptic development in male or female *Fmr1* KO mice within auditory brainstem nuclei.

**Table 5.** Statistics for two-way ANOVA to identify effects of sex and genotype

P14	VCN	MNTB	LSO
Nucleus size	Sex: *F*_1,27_ = 0.546, *p* = 0.466; genotype: *F*_1,27_ = 0.248, *p* = 0.623; interaction: *F*_1,27_ = 0.289, *p* = 0.595	Sex: *F*_1,27_ = 0.108, *p* = 0.745; genotype: *F*_1,27_ = 5.877, *p* = 0.022; interaction: *F*_1,27_ = 0.024, *p* = 0.878	Sex: *F*_1,27_ = 0.669, *p* = 0.421; genotype: *F*_1,27_ = 0.022, *p* = 0.884; interaction: *F*_1,27_ = 3.438, *p* = 0.075
Number of cells	Sex: *F*_1,27_ = 0.459, *p* = 0.504; genotype: *F*_1,27_ = 0.174, *p* = 0.680; interaction: *F*_1,27_ = 0.998, *p* = 0.327	Sex: *F*_1,27_ = 0.032, *p* = 0.860; genotype: *F*_1,27_ = 0.298, *p* = 0.590; interaction: *F*_1,27_ = 0.740, *p* = 0.397	Sex: *F*_1,27_ = 0.004, *p* = 0.947; genotype: *F*_1,27_ = 1.698, *p* = 0.204; interaction: *F*_1,27_ = 3.140, *p* = 0.088
Cell size	Sex: *F*_1,26_ = 1.106, *p* = 0.303; genotype: *F*_1,26_ = 2.796, *p* = 0.107; interaction: *F*_1,26_ = 0.086, *p* = 0.771	Sex: *F*_1,16_ = 0.225, *p* = 0.642; genotype: *F*_1,16_ = 21.198, *p* < 0.001; interaction: *F*_1,16_ = 3.111, *p* = 0.097	Sex: *F*_1,26_ = 0.009, *p* = 0.925; genotype: *F*_1,26_ = 14.746, *p* < 0.001; interaction: *F*_1,26_ = 0.392, *p* = 0.537
VGLUT	Sex: *F*_1,22_ = 4.253, *p* = 0.051; genotype: *F*_1,22_ = 0.049, *p* = 0.828; interaction: *F*_1,22_ = 0.108, *p* = 0.746	Sex: *F*_1,6_ = 1.265, *p* = 0.304; genotype: *F*_1,6_ = 3.258, *p* = 0.121; interaction: *F*_1,6_ = 5.738, *p* = 0.054	Sex: *F*_1,22_ = 0.789, *p* = 0.384; genotype: *F*_1,22_ = 0.009, *p* = 0.927; interaction: *F*_1,22_ = 0.268, *p* = 0.610
VGAT	Sex: *F*_1,22_ = 2.066, *p* = 0.165; genotype: *F*_1,22_ = 0.488, *p* = 0.492; interaction: *F*_1,22_ = 0.591, *p* = 0.450	Sex: *F*_1,6_ = 0.196, *p* = 0.674; genotype: *F*_1,6_ = 16.028, *p* = 0.007; interaction: *F*_1,6_ = 1.159, *p* = 0.323	Sex: *F*_1,22_ = 1.510, *p* = 0.232; genotype: *F*_1,22_ = 0.551, *p* = 0.466; interaction: *F*_1,22_ = 0.962, *p* = 0.337
I_SP_	Sex: *F*_1,22_ = 1.187, *p* = 0.288; genotype: *F*_1,22_ = 0.404, *p* = 0.531; interaction: *F*_1,22_ = 2.715, *p* = 0.114	Sex: *F*_1,6_ = 0.099, *p* = 0.764; genotype: *F*_1,6_ = 6.793, *p* = 0.040; interaction: *F*_1,6_ = 4.950, *p* = 0.068	Sex: *F*_1,22_ = 0.646, *p* = 0.430; genotype: *F*_1,22_ = 4.515, *p* = 0.055; interaction: *F*_1,22_ = 0.668, *p* = 0.422
Synaptophysin	Sex: *F*_1,16_ = 1.165, *p* = 0.296; genotype: *F*_1,16_ = 1.537, *p* = 0.233; interaction: *F*_1,16_ = 1.072, *p* = 0.316	Sex: *F*_1,6_ = 0.224, *p* = 0.653; genotype: *F*_1,6_ = 2.765, *p* = 0.147; interaction: *F*_1,6_ = 0.910, *p* = 0.377	Sex: *F*_1,16_ = 0.824, *p* = 0.377; genotype: *F*_1,16_ = 31.222, *p* < 0.001; interaction: *F*_1,16_ = 1.103, *p* = 0.309
IBA1	Sex: *F*_1,27_ = 1.589, *p* = 0.218; genotype: *F*_1,27_ = 1.192, *p* = 0.285; interaction: *F*_1,27_ = 2.381, *p* = 0.134	Sex: *F*_1,27_ = 0.268, *p* = 0.609; genotype: *F*_1,27_ = 0.569, *p* = 0.457; interaction: *F*_1,27_ = 0.125, *p* = 0.727	Sex: *F*_1,27_ = 0.094, *p* = 0.761; genotype: *F*_1,27_ = 0.075, *p* = 0.786; interaction: *F*_1,27_ = 1.556, *p* = 0.223
ALDH1L1	Sex: *F*_1,18_ = 0.109, *p* = 0.745; genotype: *F*_1,18_ = 4.855, *p* = 0.041; interaction: *F*_1,18_ = 3.020, *p* = 0.099	Sex: *F*_1,20_ = 0.215, *p* = 0.648; genotype: *F*_1,20_ = 1.025, *p* = 0.323; interaction: *F*_1,20_ = 0.149, *p* = 0.703	Sex: *F*_1,20_ = 0.495, *p* = 0.490; genotype: *F*_1,20_ = 4.130, *p* = 0.056; interaction: *F*_1,20_ = 2.392, *p* = 0.138

## Discussion

Previous studies of the adult *Fmr1* KO mouse brainstem reported both anatomic and functional phenotypes (Rotschafer et al., 2015; Wang et al., 2015; Garcia-Pino et al., 2017). ABR measurements showed that *Fmr1* KO mice had heightened ABR thresholds and reduced peak I, consistent with a modest hearing loss. Central auditory brainstem dysfunction in *Fmr1* KO may thus arise secondarily from reduced peripheral input. However, FMRP is expressed throughout the auditory brainstem (Beebe et al., 2014; Wang et al., 2014; Ruby et al., 2015), and thus loss of FMRP may have additional effects directly on these nuclei. Here, we examined auditory brainstem development both before and after hearing onset (Hoffpauir et al., 2006; Holcomb et al., 2013) to determine to when these phenotypes emerge.

### Smaller neurons in auditory nuclei of Fmr1 KO mice during postnatal development

We found broad variability among nuclei in the emergence of differences in cell size. Differences in VCN cell size were not present at any of the ages examined, and thus develop after P14. As in adults, MNTB neurons in *Fmr1* KO mice were smaller than in wild type at early postnatal ages. In contrast to VCN, these differences were already seen at P1, before hearing onset and before the onset of spontaneous activity (Wang and Bergles, 2015; Sierksma et al., 2017). FMRP might thus have a cell-autonomous function that regulates cell size independent of levels of synaptic input.

Our data show that a medial–lateral gradient in cross-sectional cell area was evident to a moderate degree at early ages in wild-type mice and became more pronounced at P14. In contrast, the cell size gradient did not form in *Fmr1* KO mice until after hearing onset. The delay in the formation of a cell size gradient in MNTB suggests a role for FMRP. FMRP is an activity-dependent negative inhibitor of mRNAs that is expressed in a medial-to-lateral gradient within MNTB (Ruby et al., 2015) and plays an important role in maintaining protein expression gradients in MNTB (Bassell and Warren, 2008; Strumbos et al., 2010). FMRP regulates expression of the potassium channel kv3.1b, which is expressed in a parallel gradient, and loss of *Fmr1* results in a loss of this kv3.1b gradient (Strumbos et al., 2010). It is thus feasible that the gradient of FMRP expression, through a variety of downstream targets, contributes to the formation of the gradient of cell size. However, the eventual emergence of a size gradient in mutants suggests that FMRP is not needed for the maintenance of this gradient. A previous study demonstrated that auditory evoked activity is needed to maintain this gradient (Weatherstone et al., 2017). Taken together with our current findings, these results suggest distinct mechanisms for the establishment versus the maintenance of gradients in MNTB cell size.

In LSO, we found significant reductions in cell size in *Fmr1* KO mice at all ages examined. Because these differences are not observed in adults (Rotschafer et al., 2015), our results suggest that they resolve by the time mice reach adulthood. In *Fmr1* KO mice, synaptic strengthening events are delayed, but are considerably larger than those in wild-type mice (Garcia-Pino et al., 2017), and the adult *Fmr1* KO LSO receives an exaggerated amount of input (Rotschafer et al., 2015; Garcia-Pino et al., 2017). Working in concert, these factors may overcome the early phenotype of smaller cells in *Fmr1* KO mice.

The reduction in cell size that we observed in the early postnatal MNTB and LSO were not accompanied by any differences in the nucleus size or cell number. These observations suggest that the nuclei contain greater intercellular space. The increased number of astrocytes in LSO might fill some of the space. In MNTB, where this issue persists through adulthood, this space might be filled with expanded axon tracts in the ventral acoustic stria, changes in the extracellular matrix, and/or increased neuropil volume.

### Synaptic protein expression is altered in MNTB in Fmr1 KO mice

Changes in neuronal excitability contribute to neurologic dysfunction in FXS (Contractor et al., 2015). In the auditory brainstem, where synaptic balance is a key factor in sound processing and sound localization (Tollin, 2003), increased excitability could lead to hyperacusis and difficulties in sound localization. Indeed, *Fmr1* KO mice have shifted sensitivity for interaural level differences (Garcia-Pino et al., 2017). Enhanced gain leading to hyperacusis in FXS may originate, at least in part, in the auditory brainstem nuclei. The increase in VGAT in MNTB, a sign-inverting relay nucleus, could lead to enhanced excitation in targets of MNTB (Rotschafer et al., 2015); additionally, increased excitation in LSO has also been shown to arise from VCN (Garcia-Pino et al., 2017). Both of these observations suggest that the superior olivary complex may increase gain in the auditory pathway in *Fmr1* KO mice.

In MNTB, increased VGAT expression in *Fmr1* KO mice was seen at P6 and persisted into adulthood. The I_SP_ ratios in MNTB are generally large and positive, reflecting the presence of the large, excitatory input from the calyx of Held. Ratios are reduced in magnitude but remain positive in *Fmr1* KO mice relative to wild-type mice. At P14, the I_SP_ ratios for both genotypes were somewhat higher than those previously published in adult mice (Rotschafer et al., 2015), suggesting that this phenotype continues to develop with synaptic maturation after hearing onset. The source of these increased VGAT terminals is likely from VNTB, the principal source of inhibitory input to MNTB (Albrecht et al., 2014). However, a previous study did not find any difference in GAD67 levels in the VNTB or in other auditory brainstem nuclei (McCullagh et al., 2017) in adult *Fmr1* KO animals. This finding suggests that the mutation might lead to enhanced branching of the normal population of VNTB GAD67-positive cells to result in more exuberant terminals in MNTB in *Fmr1* KO mice. In that study, a decrease in expression of the glycine transporter Glyt2 was seen, but only in the medial, high-frequency region of the MNTB. It is not known whether the shift from GABA to glycine is altered in *Fmr1* KO mice. However, our results suggest that altered inhibitory input to MNTB is seen before hearing onset, before inhibition has matured in the auditory brainstem.

Our data suggest that increased VGAT expression drives the decrease in I_SP_ in *Fmr1* KO mice. There is other evidence, however, that calyx of Held volume may also grow larger in *Fmr1* KO mice (Wang et al., 2015). Our interpretation is based on VGLUT2 expression only, and other excitatory presynaptic proteins may be affected in *Fmr1* KO mice. Interestingly, despite an enlarged presynaptic terminal, cells in the *Fmr1* KO MNTB did not show significant differences in firing behavior (Wang et al., 2015), presenting the possibility that a larger calyx of Held may be countered by an increase in presynaptic inhibitory input in *Fmr1* KO. While MNTB cells may have larger calyces of Held, work done in *Fmr1* KO rats found fewer MNTB neurons received input from VCN globular bushy cells (Ruby et al., 2015).

Previous studies demonstrated an increase in VGLUT expression in the adult LSO (Rotschafer et al., 2015; Garcia-Pino et al., 2017), as well as an increase in VGAT (Rotschafer et al., 2015). Although neither VGLUT nor VGAT differences were noted in LSO during postnatal development, we found a significant increase in synaptophysin, a broad marker for presynaptic terminals, in LSO in *Fmr1* KO mice at both P6 and P14. This observation suggests that overall input is increased. The I_SP_ ratio was significantly lower in mutants than in wild-type mice at P14; this difference is not evident in adults (Rotschafer et al., 2015). Interestingly, no differences were seen between wild-type mice and *Fmr1* KO mice in adult LSO using GlyT2 as a marker of inhibitory input (Garcia-Pino et al., 2017). While VGAT is expressed in both GABAergic and glycinergic inputs (Dumoulin et al., 1999), these findings highlight the possibility that loss of FMRP might differentially affect the expression of these presynaptic markers.

### Glial cells in Fmr1 KO auditory brainstem nuclei

Microglia have several roles in synaptic maturation, neuronal homeostasis, and immune responses (Hanisch, 2002, 2013; Hanisch et al., 2004). Notably, phagocytotic and neuroprotective functions of microglia are impaired in various autism spectrum disorders (Arcuri et al., 2017; Lee et al., 2017). Astrocytes are known to shape synapses and dendritic arbors; wild-type hippocampal neurons cultured with *Fmr1* KO astrocytes adopted a dendritic morphology similar to that found in *Fmr1* KO mice (Jacobs et al., 2010, 2016).

Typically, mice steadily gain both microglia and astrocytes in the auditory brainstem between P0 and P14 (Dinh et al., 2014) Microglia and astrocytes can be found in close apposition to developing calyces in MNTB (Dinh et al., 2014). We found that microglia develop normally in the auditory brainstem of *Fmr1* KO mice, but that astrocytes are elevated in VCN and LSO at P14. Microglia were positively correlated with VGLUT in the P6 MNTB in *Fmr1* KO mice. Astrocytes were strongly positively correlated with both VGLUT and VGAT in P14 in LSO in *Fmr1* KO mice, but not in wild-type mice. The findings suggest that glial cells represent an important factor in establishing excitatory and inhibitory synapses and that their role in synaptic development may be enhanced in FXS.

### Lack of sex differences

FXS is far more common and severe in males, as they lack an additional X-chromosome that may partially compensate for *Fmr1* inactivation (Inaba et al., 2013; Godler et al., 2015). The female *Fmr1* KO mice used here contain two alleles of the *Fmr1* KO, one on each X-chromosome. Male and female *Fmr1* KO mice show differences in behavioral measures (Nolan et al., 2017), vocalization production (Reynolds et al., 2016), and neurophysiology (Giráldez-Pérez et al., 2013; Lokanga et al., 2014; Scremin et al., 2015), but other studies showed similar behaviors between the sexes (Ding et al., 2014). We found that the auditory brainstem phenotypes we observed did not differ between males and females, suggesting predominantly common molecular mechanisms that are not selectively altered by the mutation.

## Conclusion

In addition to enhanced responses to sensory stimuli, individuals with FXS have a host of symptoms that are confined to childhood (reviewed in (Turk, 2011). This invites the question of whether the symptoms of FXS result from disrupted brain development during critical periods, or from a general and ongoing effect of loss of FMRP. Here, we found delays in auditory brainstem nucleus development and imbalances in synaptic input, which potentially contribute to the hyperacusis found in adult *Fmr1* KO mice. Our findings show developmental effects at very young ages, implying that early developmental events initiate some of the auditory phenotypes in FXS.

## Synthesis

Reviewing Editor: Tallie Z. Baram, University of California-Irvine Decisions are customarily a result of the Reviewing Editor and the peer reviewers coming together and discussing their recommendations until a consensus is reached. When revisions are invited, a fact-based synthesis statement explaining their decision and outlining what is needed to prepare a revision will be listed below. The following reviewer(s) agreed to reveal their identity: Roland Bender.

The manuscript is acceptable as is.
